# Viral Single-Strand DNA Induces p53-Dependent Apoptosis in Human Embryonic Stem Cells

**DOI:** 10.1371/journal.pone.0027520

**Published:** 2011-11-17

**Authors:** Matthew L. Hirsch, B. Matthew Fagan, Raluca Dumitru, Jacquelyn J. Bower, Swati Yadav, Matthew H. Porteus, Larysa H. Pevny, R. Jude Samulski

**Affiliations:** 1 Gene Therapy Center, University of North Carolina, Chapel Hill, North Carolina, United States of America; 2 Department of Pharmacology, University of North Carolina, Chapel Hill, North Carolina, United States of America; 3 Department of Genetics, University of North Carolina, Chapel Hill, North Carolina, United States of America; 4 Human Embryonic Stem Cell Core Facility, University of North Carolina, Chapel Hill, North Carolina, United States of America; 5 Department of Cell and Developmental Biology, Neuroscience Center, University of North Carolina, Chapel Hill, North Carolina, United States of America; 6 Department of Pathology and Laboratory Medicine, University of North Carolina, Chapel Hill, North Carolina, United States of America; 7 Department of Pediatrics-Cancer Biology, Stanford University, Palo Alto, California, United States of America; University of Southern California, United States of America

## Abstract

Human embryonic stem cells (hESCs) are primed for rapid apoptosis following mild forms of genotoxic stress. A natural form of such cellular stress occurs in response to recombinant adeno-associated virus (rAAV) single-strand DNA genomes, which exploit the host DNA damage response for replication and genome persistence. Herein, we discovered a unique DNA damage response induced by rAAV transduction specific to pluripotent hESCs. Within hours following rAAV transduction, host DNA damage signaling was elicited as measured by increased gamma-H2AX, ser15-p53 phosphorylation, and subsequent p53-dependent transcriptional activation. Nucleotide incorporation assays demonstrated that rAAV transduced cells accumulated in early S-phase followed by the induction of apoptosis. This lethal signaling sequalae required p53 in a manner independent of transcriptional induction of Puma, Bax and Bcl-2 and was not evident in cells differentiated towards a neural lineage. Consistent with a lethal DNA damage response induced upon rAAV transduction of hESCs, empty AAV protein capsids demonstrated no toxicity. In contrast, DNA microinjections demonstrated that the minimal AAV origin of replication and, in particular, a 40 nucleotide G-rich tetrad repeat sequence, was sufficient for hESC apoptosis. Our data support a model in which rAAV transduction of hESCs induces a p53-dependent lethal response that is elicited by a telomeric sequence within the AAV origin of replication.

## Introduction

It is becoming increasingly appreciated that human embryonic stem cells (hESCs) have an altered DNA damage response compared to multipotent and differentiated cells: i) hESCs display high rates of spontaneous apoptosis and induce rapid apoptosis in response to, generally, sub-lethal forms of DNA stress (1), ii) apoptotic induction in hESCs is often elicited via a p53-transcription independent mitochondrial pathway [1], [2], iii) hESCs are deficient in p21 abundance despite significant p53 transactivation of the p21 promoter upon DNA stress [3] and iv) hESCs may display unique cell-cycle checkpoint kinetics in response to ionizing radiation [3]. These characteristics help to define/maintain the pluripotent versus differentiation status of hESCs, maintained in part and also characterized by micro RNA profiles [4]. Furthermore, such intolerance to genotoxic stress is likely a mechanism to purge genetic abnormalities [3].

Natural insults that induce cellular DNA damage responses include single-strand DNA viruses, such as the *Parvoviridae* members B19, minute virus of mice and adeno-associated virus (AAV) in manners both dependent and independent of viral gene expression [5], [6]; [ reviewed in 7]. In particular, AAV is a small (25 nm) non-enveloped virus of the family *Parvoviridae* genus *Dependovirus*. The protein capsid is packaged with a 4.7 kb single-strand DNA genome flanked at both ends by 145 nucleotide (nt) inverted terminal repeats (ITRs) that are necessary for the initiation of replication and packaging, among other processes (reviewed in [8]). An 80 nt sequence within the AAV ITR containing the replication protein (Rep) binding site and the terminal resolution site is sufficient for these aspects of the viral life cycle [9]. This viral telomere sequence shares characteristics of human telomeres including existing as ssDNA and having G-rich repeated elements. For transducing vector applications, we developed recombinant AAV (rAAV) in which all viral genes are replaced by a sequence of choice such that only the AAV ITRs remain [10]. Such vectors have demonstrated success for gene delivery applications in cell culture, in animal models, as well as for human disease therapy. In such instances, the majority of transgenic DNA is converted to double-strand monomer circles and concatemers for episomal persistence, processes stimulated by the ITRs [11], [12].

It is well documented that AAV infection of dividing cells in culture results in a DNA damage response including cell cycle checkpoint activation [13], [14], [15]. This response was found independent of the expression of viral proteins and, instead, is attributed to the ITR sequence [5]. Transduction of normal dividing cultured cells results in G2 arrest, followed by normal cycling thereafter [5]. This cell cycle perturbation is dependent upon the activity of p53 and its downstream regulatory cascades. In fact, in the absence of a functional p53-p21-pRb signaling cascade, a deficiency associated with many cancers, cells do not maintain the G2/M checkpoint, and undergo AAV-induced apoptosis upon transduction [3], [5].

This work investigated the ability of 9 AAV serotypes to transduce hESCs of different origins. However, a previously undescribed apoptotic phenotype was observed that directly correlated with the level of transduction. The rAAV-induced apoptosis was mediated at the level of a lethal DNA damage response as demonstrated by p53ser-15 phosphorylation, increased gamma-H2AX and p53-dependent trans-activation of the p21 promoter. Consistently, AAV protein capsids without DNA were well tolerated by hESCs. Further investigation into this lethal DNA damage response implicated a 39 nucleotide G-rich telomere-like tetrad repeated sequence within the ITR as the apoptotic trigger. An oligonucleotide with the sequence, but not the reverse complement sequence, formed intermolecular interactions using native gel electrophoresis suggestive of G-quadruplex formation. Collectively, these results demonstrate that rAAV transduction of hESCs induces a p53-dependent lethal DNA damage response in a manner reminiscent of G-quadruplex induced apoptosis.

## Results

### AAV transduction of hESCs

**Figure 1:**
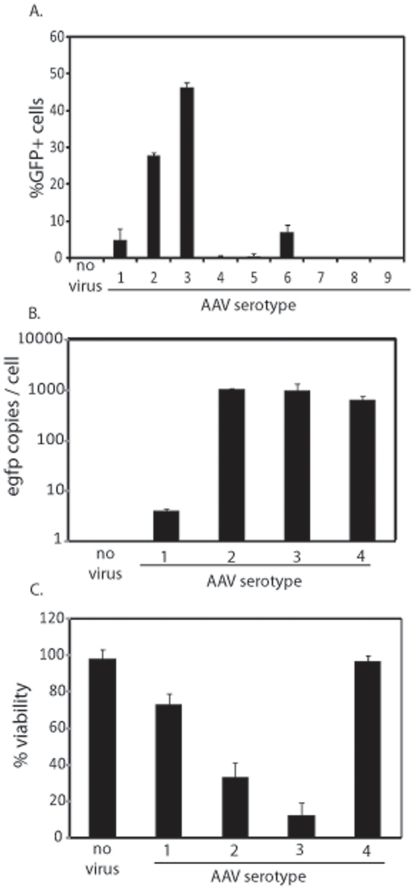
Recombinant AAV Transduction of hESCs. The indicated AAV serotypes were packaged with a self-complementary CMV-*egfp* genome and used to infect human embryonic stem cells (hESCs) at 100,000 viral genomes per cell. 24 h post-infection (post-infection) cells were harvested and GFP+ cells were quantitated by flow cytometry (A.). Treated cells were also analyzed for intracellular transgene copy number normalized to the lamin B gene (B). Cell viability was also measured under the indicated conditions via dye exclusion (C) and significant decreases (p-value<0.05) were noted in all cases (compared to no virus) except with AAV4 treatment (p-value = 0.44). The results are averaged from at least 6 replicates for each treatment group and the standard deviation is depicted.

Embryonic stem cell therapies are currently being developed for therapeutic applications and have already entered the clinic for the treatment of multiple diseases. To investigate the efficiency of rAAV transduction as a tool by which hESCs may be genetically manipulated for use in clinical therapy, capsid serotypes 1–9 packaged with a self-complementary (sc) CMV-*egfp* reporter cassette were initially used at a multiplicity of infection (MOI) of 100,000 (viral genomes/cell). Of the analyzed serotypes, AAV3B demonstrated the highest transduction at 46% GFP^+^ cells after 24 h (Figure 1A). AAV2, AAV6, and AAV1 were also capable of hESC transduction, albeit at lower efficiencies whereas all other serotypes demonstrated transduction efficiencies of less than 1% report (Figure 1A).

To determine if the differential expression of the *egfp* transgene among the most efficient serotypes directly correlated with viral gene copy number/cell, total DNA was extracted (including that from intact intracellular AAV particles) and quantitated by PCR (Q-PCR). The results were normalized to the copy number of the human lamin B2 gene and are presented as viral genomes/cell. In general, the copy number of the *egfp* transgene directly correlated with the percentage of GFP^+^ cells determined by flow cytometry (Figure 1B). However, there was an exception, hESCs treated with rAAV4 demonstrated no GFP+ cells, yet the intracellular transgene copy number was equivalent to that of rAAV3B transduced hESCs (Figure 1A, B). This observation suggests that the AAV4 capsid is capable of cell entry but is deficient for trafficking/uncoating in hESCs.

Of particular note during the these experiments is that at the tested time point, most of the GFP+ cells had detached from the fibronectin coated plates and were compromised for membrane integrity (Figure 1C). In fact, by 72 h post-infection all GFP+ cells had lost viability, an effect that was observed in hESCs of different origin (WiCell H1, H7, H9 and CBh6). It is important to note, that the AAV-induced toxicity was independent of the vector production method (different chromatographies or cesium chloride gradient centrifugation), the dialysis buffer, the GFP protein, and AAV particle purity was deemed high by electron microscopy (unpublished data).

### AAV induces apoptosis in hESCs

**Figure 2:**
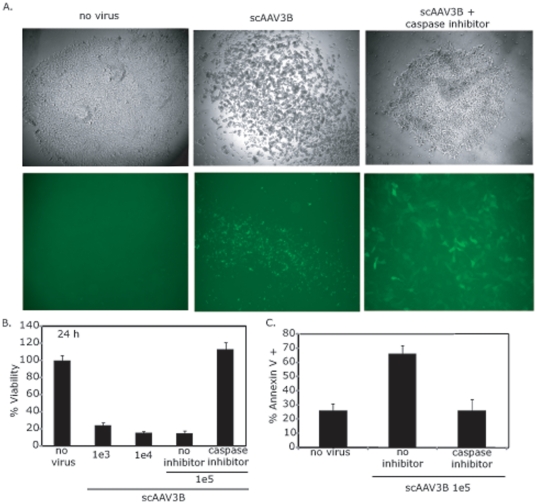
Recombinant AAV Transduction of hESCs induces Apoptosis. Human embryonic stem cells (hESCs) were treated with 100,000 AAV3B particles packaged with a self-complementary CMV-*egfp* genome. 24 h post-infection (p.i.) cellular morphology and GFP fluorescence was observed by microscopy in the presence or absence of a pan-caspase inhibitor (A). Cell viability of hESCs treated with the indicated amount of the scAAV3B-*egfp* vector described above was analyzed by dye exclusion 24 h post-infection (B). Significant decreases (p-value<0.05) were noted in all cases except when the caspase inhibitor was used (p-value>0.1). hESCs treated as described were analyzed for annexin V staining 8 h post-infection (C). A significant increase (p-value<0.05) was noted for cells treated with only rAAV3B. The data in (B) and (C) represents the average from at least 6 replicates per treatment group and the standard deviation is depicted as well.

The dose-dependent toxicity of scAAV3B-*egfp* was evaluated over time, and morphological changes characteristic of apoptosis (small, spherical, loss of matrix adherence) were observed as early as 8 h after rAAV infection. At 24 h post-infection, hESC viability was assessed by microscopy and quantitated by dye exclusion (Figure 2A, B). At this time point, over 70% of cells were compromised for membrane integrity, compared to vehicle only (PBS) treatment controls (labeled no virus), at both tested MOIs (Figure 2A, B).

To confirm that the rAAV-induced toxicity observed in hESCs is characteristic of apoptosis, a variety of assays were employed. First, the ability of scAAV3B-*egfp* to induce cell death was examined in hESCs cultured in the presence of the pan-caspase inhibitor, Z-VAD-FMK. Upon incubation with Z-VAD-FMK, the toxic phenotype was completely abrogated 24 h post-infection (Figure 2). As a second method to determine the mechanism of cell death, membrane changes characteristic of apoptosis were evaluated by annexin V staining 8 h after scAAV3B vector addition. At this time point, annexin V staining increased 3-fold in cells treated with rAAV, an effect that was completely eliminated by caspase inhibition (Figure 2C). Chromosome condensation was evaluated by DAPI staining as a third and final method to determine whether the hESCs were undergoing apoptosis in response to rAAV transduction. Eight hours after scAAV3B infection, hESCs demonstrated a dramatic increase in punctate nuclear staining compared to cells that were not treated (Figure S1). Collectively these results demonstrate that the observed hESC toxicity induced by rAAV transduction is apoptosis.

To determine if the rAAV-induced apoptosis was specific to the WiCell H9 line, CBh6, WiCell H7s, and H1 cells were also evaluated. rAAV transduction videos of WiCell H7s, H9s and CBh6 hESCs demonstrating this phenomenon can be found at http://genetherapy.unc.edu/samulski.htm. For instance, H1 cells displayed a GFP^+^ phenotype 24 h post-infection by scAAV3B-*egfp* (MOI 1e5; Figure S2A). However, in contrast to the results using the H9s, the onset of complete rAAV-induced apoptosis was delayed in the H1s with only a 2-fold decrease in viability 24 h post-infection followed by near complete toxicity during the next 3 days (Figure S2B). These results suggest that the kinetics of rAAV-induced apoptosis in hESCs may correlate with the cell cycle kinetics of the particular hESC. Additionally, the H1 cells exhibit decreased abundance of the Oct4 transcript (relative to GAPDH), a decreased mitotic entry rate, and a lower S-phase fraction when compared to the H9s (Figure S2 C, D).

To demonstrate that the rAAV vector preps do not induce indiscriminate apoptosis, and that this phenomenon is unique to pluripotent cells, rAAV transduction of partially differentiated hESCs was investigated. hESCs were grown to embryoid bodies and differentiated towards a neural lineage for 15 days. The same scAAV3B-*egfp* vector preparation previously shown to induce apoptosis in hESCs was used to infect the differentiated cells (MOI of 100,000). At 24 h post-infection, the majority of the differentiated cells demonstrated a GFP^+^ phenotype with negligible toxicity consistent with a previous report (Figure S3; 25). This result is consistent with a previous report on the etoposide-induced DNA damage response in pluripotent hESCs versus hESCs in various stages of differentiation [3].

### Recombinant AAV Transduction induces a DNA damage response in hESCs

**Figure 3:**
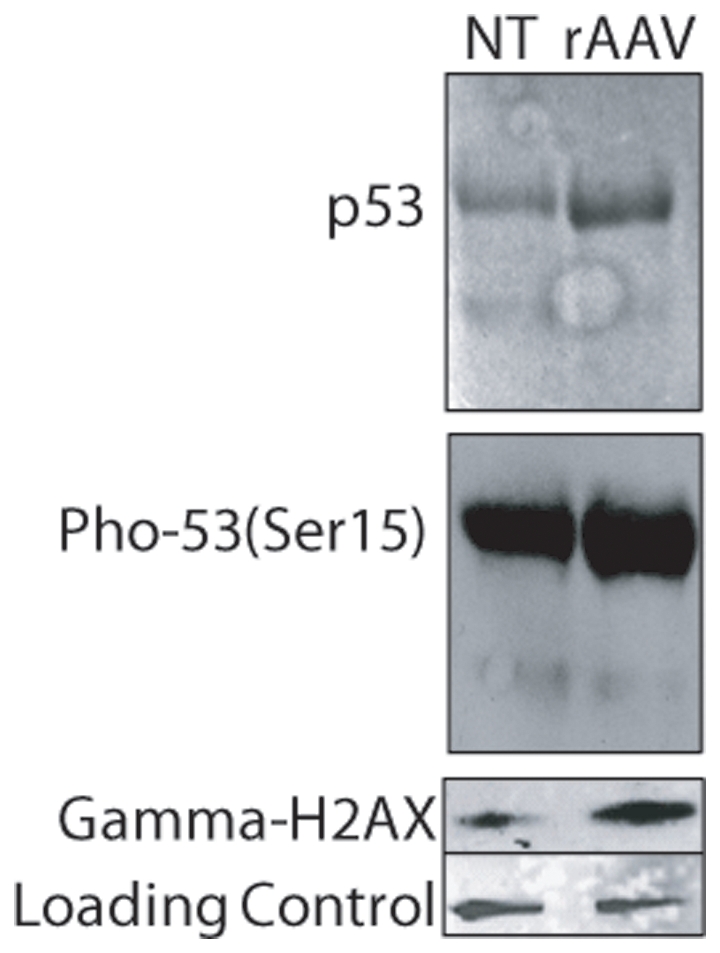
Recombinant AAV Transduction Activates a DNA Damage Response in hESCs. Total protein from scAAV3b-*egfp* transduced hESCs was harvested 8 h post-infection. Western blotting analysis was performed using the indicated antibodies.

The TP53 gene product, p53, plays an integral role in coordinating the cellular response to damaged DNA, including cell cycle arrest and the induction of apoptosis. Upstream sensors, such as ATM and DNA-PK, activate p53 following DNA damage, in part, by phosphorylation at serine 15 (ser-15), a phenomenon reported for both differentiated cells and hESCs [3]. Therefore, to investigate whether a DNA damage response was induced in hESCs upon rAAV transduction, we analyzed total p53 abundance and the phosphorylation status of ser-15 of p53 (Figures 3, S5). Total protein from hESCs treated with scAAV3B-*egfp* (MOI of 100,000) was analyzed by Western blot 8 h post-infection. The results demonstrate a dramatic increase in the phosphorylation of p53ser-15 for rAAV transduced cells while no phosphorylation at that site was observed for untreated cells (Figure 3). Another indicator of damaged DNA, phosphorylation of ser-139 on H2AX (gamma-H2AX), was also investigated. Increased gamma-H2AX was observed for hESCs treated with AAV3B-*egfp* for 8 h (Figure 3). Collectively, the results thus far demonstrate that rAAV transduction of hESCs induces a DNA damage response that concludes in apoptosis.

### Recombinant AAV-induced hESC apoptosis is p53-dependent

**Figure 4:**
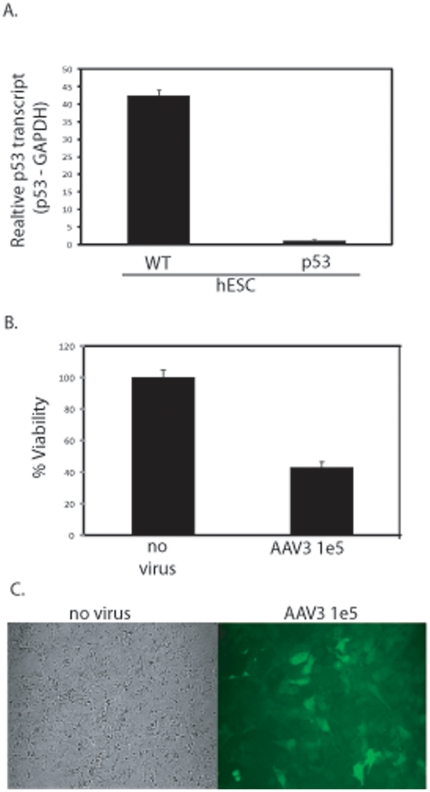
Recombinant AAV-Induced hESC Apoptosis is p53-Dependent. A polyclonal p53 deficient H9 hES cell line was constructed using lenti-viral transduction of a p53-specific shRNA cassette. After selection, total RNA was harvested, converted to cDNA and the relative abundance of p53 transcript, compared to the GAPDH housekeeping transcript, was determined by Q-PCR (A). The parental H9 hESCs were used as a wild type control (A). scAAV3B-*egfp* transduction of p53 deficient hESCs at a MOI of 100,000 was performed. 24 h post-infection viability was determined by dye exclusion (B) microscopy (C). The results in (A) and (B) are averaged from at least 6 replicates for each treatment group and the standard deviation is depicted. The differences noted in both (A) and (B) have p-values<0.005).

As p53 has been reported necessary for the induction of apoptosis in hESCs in response to UV-induced DNA damage and etoposide [2], its role in rAAV-induced apoptosis was investigated. First, a p53 knockdown polyclonal hESC line was generated using lenti-viral vectors to deliver constitutively expressed p53 shRNA. The knockdown of p53 transcript was confirmed using Q-PCR of cDNA and normalized to the GAPDH transcript. The polyclonal p53 shRNA H9 population demonstrated a 45-fold reduction in the p53 transcript (normalized to GAPDH) compared to the wt parent (Figure 4A). In addition, a marked decrease in p53 protein abundance was also observed (Figures S4A, S5) This H9 cell population (referred to as hESC/p53-) maintained near identical levels of Oct4 transcription as determined by Q-PCR compared to the wt parent (Figure S4B). Next, these cells were transduced by scAAV3B-*egfp* (MOI = 100,000) and harvested for determination of cell viability 24 h post-infection. At this time point there was a 2-fold decrease in cell viability for rAAV treated hESC/p53- cells compared to a 10-fold decrease for wild type hESCs when compared to their respective untreated control (Figures 2B and 4B). Additionally, transduction (GFP+ phenotype) was scored in nearly all scAAV3B-*egfp* treated hESC/p53- cells with retention of normal hESC morphology (Figure 4C). After 5 days post-infection, a time point when all transduced wild type hESCs are dead, the majority of p53 deficient hESCs survive and maintain productive transduction as evidenced by a GFP+ phenotype (Figure S4C).Collectively, these data demonstrate that the apoptotic DNA damage response induced by rAAV is dependent upon p53.

### p53 trans-activation following Recombinant AAV transduction

**Figure 5:**
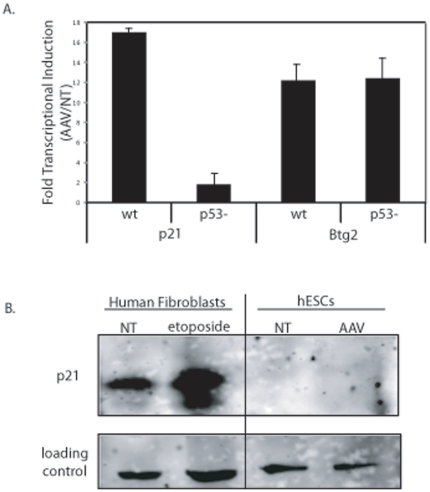
p53 Transactivation Occurs Following Recombinant AAV Infection. (A) Fold induction of p21 and Btg2 transcripts via quantitative PCR following rAAV infection. Data is presented as the value determined for hESCs infected with 100,000 AAV3B vectors 8 h post-infection divided by the transcript amount for hESCs not treated. Values were determined in both H9s and H9s with p53 knocked down as described in text (p53-). A significant difference was noted only for p21 transcript levels in the absence of p53 (p-value<0.005). B) Western blot analysis of p21 abundance following rAAV infection (8 h post-infection). Human fibroblasts treated with the DNA damaging agent etoposide was used as an antibody control. The results are averaged from at least 3 replicates for each treatment group and the standard deviation is depicted.

p53 functions as a transcriptional regulator of genes involved in cell cycle control and apoptosis, among other processes. Therefore, we analyzed transcript abundance following rAAV transduction for the cyclin dependent kinase inhibitor 1A (p21), Btg2, Bcl-2, Bax, and Puma. The p21 and Btg2 transcripts both increased >10-fold 8 h post-infection (Figure 5A). The cellular response for these two transcripts in the hESC/p53- cells was also evaluated in the same manner. The results demonstrate that the 17-fold increase in p21 transcription was p53-dependent while the increase in Btg2 transcription was p53-independent (Figure 5A). Given that rAAV-induced apoptosis is dependent on p53 (Figure 4B, C), and p21 transcriptional induction is p53-dependent (Figure 5A), p21 abundance was directly evaluated by Western blot analysis. Despite our successful detection of p21 in our positive control cells (human fibroblasts), no p21 was detected in the hESCs despite the 17-fold induction of transcription following AAV infection (Figure 5B). This result is consistent with barely detectable levels of p21 protein in hESCs despite a UV-induced stimulation of p21 transcription [2]. No significant change in transcript abundance was noted for promoters of genes involved in regulation of apoptosis (Bcl-2, Bax, Puma; Figure S6). This suggests that the apoptotic response in rAAV transduced hESCs is mediated by p53 in a transcription independent manner.

### Recombinant AAV infection impairs S-phase progression in hESCs

**Figure 6:**
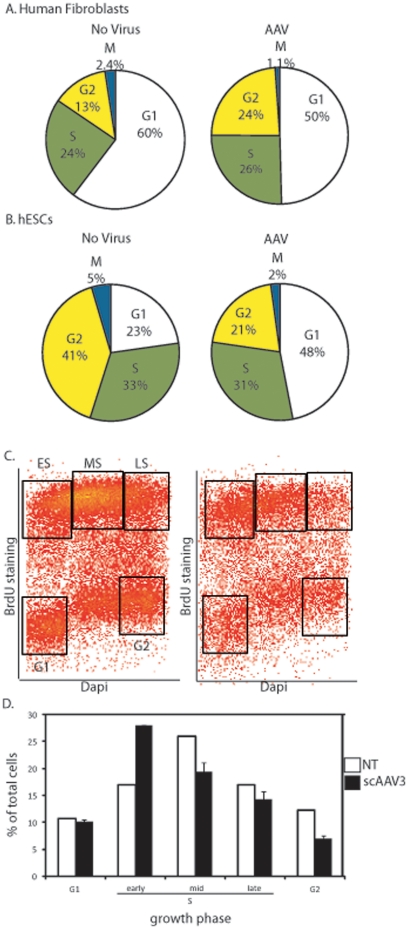
AAV Infection of hESCs Induces Early S-phase Accumulation. A) Normal human fibroblasts treated with 100,000 AAV3B vectors were treated 6 h post-infection with nocodazole. 2 h later cells were harvested and stained with DAPI and a mitotic marker as described in the text. Flow cytometry allowed the determination of cells in the indicated growth phases; however by this assay early S-phase and G1 cells are not distinguished. B) The method described above was performed on hESCs and processed in the same manner. C) hESCs treated with AAV as described above were given EdU 6 h post-infection. 2 h later cells were harvest, stained with DAPI and analyzed using flow cytometry. A representative dot plot is shown with the boxes drawn to represent different growth phases. D) Average quantitation of cells depicted in C). Collectively, the results are averages from at least 3 independent experiments and the standard deviation is depicted (* indicates p-value<0.005).

The p21 gene product regulates cell cycle progression at the G1 checkpoint via direct inhibition of cyclin-CDK2 and cyclin-CDK4 activities [16]. However, the role of p21 in hESCs remains largely unknown as protein levels are barely detectable and the presence of a G1/S checkpoint in hESCs is debatable [2], [17], [18]. To investigate the hESC cell cycle response following rAAV transduction, cell cycle kinetics were examined after a 2 hr nocodazole treatment which prevents mitotic exit. Cells were stained with an MPM-2 antibody, a specific marker of mitosis, and DAPI to measure cellular DNA content [35]. Samples were then analyzed by flow cytometry and MPM2^+^ cells with 4N DNA content were considered mitotic. Immortalized human diploid fibroblasts, which tolerate rAAV transduction, were initially investigated after vector treatment and displayed an increased fraction of cells in G_2_ phase, consistent with previous reports (Figure 6A) [5]. In contrast, rAAV transduced hESCs displayed increases in what appears to be the G_1_ fraction 8 h after rAAV infection by this assay (Figure 6B). Since the measurement of DNA content alone cannot accurately distinguish between true G_1_ cells and very early S-phase cells, EdU incorporation was measured after rAAV infection to identify hESCs undergoing DNA replication. The hESCs entered and progressed through S-phase as exhibited by the incorporation of EdU evenly across cells with 2N-4N DNA content (Figure 6C, D). In contrast, the fraction of cells in early S-phase after scAAV3B-*egfp* transduction increased almost 2-fold over the non-infected hESCs (Figure 6C, D). Taken together, these results suggest that transduced hESCs readily enter S-phase and subsequently, undergo apoptosis [5].

### The AAV origin of replication induces apoptosis in hESCs

**Figure 7:**
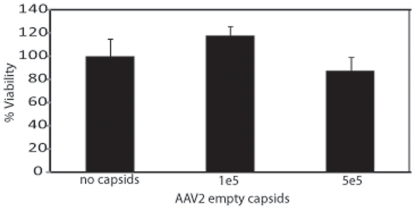
AAV empty capsid transduction of hESCs is non-toxic. AAV2 capsids were administered to hESCs at the indicated dose. Cell viability was determined after 24 h by dye exclusion. The average of at least 6 replicates is presented with the standard deviation. No significant difference (p-value>0.1) was noted when treatment groups were compared to the no capsid control).

Since rAAV infection has been previously regarded as non-toxic in many other cell types, it was important to delineate the mechanism by which rAAV induces apoptosis. The results above clearly demonstrate an apoptotic DNA damage response upon rAAV infection suggesting the single-strand DNA is toxic. However, it remains a formal possibility that the protein capsid is involved in the apoptotic response and therefore, we treated hESCs with empty rAAV capsids. In these experiments, empty AAV2 particles were utilized instead of AAV3B particles, since an antibody to intact AAV2 particles exists and can be used to determine particle number. Furthermore, previous work from our laboratory has demonstrated that empty AAV2 particles efficiently enter Hela cells and accumulate peri-nuclear, similar to the majority of genome containing capsids [19]. No significant toxicity was observed for AAV2 empty capsid treated hESCs, even at very high particle numbers (Figure 7). In contrast, dramatic toxicity was observed for hESCs treated with scAAV2-*egfp* (Figure 1C). This result suggests that receptor binding, virus particle uptake, endosomal trafficking, and peri-nuclear accumulation do not induce the observed apoptosis in hESCs.

**Figure 8:**
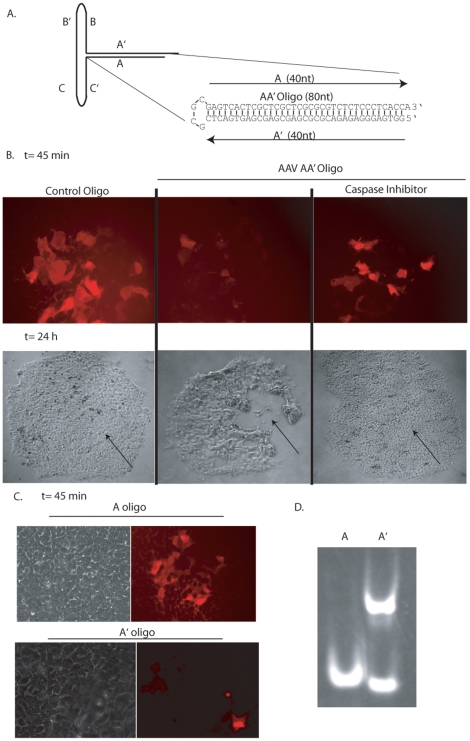
AAV Inverted Terminal Repeats Induce Apoptosis in hESCs. A) Cartoon depiction of the AAV inverted terminal repeat. The experimental oligonucleotides are also depicted as AA' (80-mer), A (39-mer) and A' (39-mer). B) hESCs were injected with AA' or a control oligonucleotide with no AAV sequence under the indicated conditions. The DNA solution contained rhodamine allowing visualization of injected cells (red). Microscopy was performed at the indicated time points. C) DNA microinjections of the A or A' oligonucleotides as described above. D) Native gel analysis of the A and A' oligonucleotides as described in the text.

Since the transgene product (GFP) and the AAV capsid were not involved in hESC apoptosis (Figure 7, unpublished data), it was hypothesized that the AAV genomic elements might be responsible for the apoptosis observed in hESCs. In fact, a previous report demonstrated that the AAV ITR sequence induces apoptosis in p53-deficient cancer cells [5]. To investigate whether the AAV ITR is toxic in hESCs we performed DNA microinjections using oligonucleotides having AAV ITR homology. In preliminary experiments, we observed that the entire 145 nt ITR sequence (Figure 8A) induces rapid hESC apoptosis. We then reduced the AAV ITR by elimination of the BB', CC', and DD' elements, while retaining only the AA' sequences (AA'oligo, Figure 8A). This sequence contains the AAV Rep binding element, a GC-rich telomeric tetrad repeat which is among the minimal elements necessary for rAAV replication. The 80 nt DNA oligonucleotide exhibiting the AAV AA' sequence was microinjected into hESCs along with a labeling dye. Two hours post-injection, the majority of cells receiving the AA' oligonucleotide underwent apoptosis, while cells microinjected with a sized-matched control oligonucleotide, containing no ITR homology, were healthy in appearance (Figures 8B, C, S7). The toxicity induced by the AA' oligonucleotide was completely abrogated in cells grown in caspase inhibitor 1 h prior to injection and thereafter (Figures 8B, C, S7). No lethal bystander effect was noted for cells neighboring those injected with the ITR oligonucleotide. The AA' oligonucleotide was then dissected into 2 complementary oligos, A and A', both 39 nt in length. Remarkably, the 2 oligonucleotides induced contrasting phenotypes when injected into hESCs; the A oligo was well tolerated and did not induce significant toxicity whereas the A' induced complete toxicity a few hours following the injections (Figure S7). It is known that 3′ strands of human telomeres contain short G-rich sequences that exist as single-strand overhangs similar the AAV ITRs. These telomeric regions often adopt higher order structures known as G-quartets which can stack to form G-quadruplexes via inter- or intra-molecular binding (reviewed in [20]). Additionally, such structures are involved in stalled replication forks, apoptotic signaling and the formation of these structures are often analyzed by native gel electrophoreses [21]. The A oligo migrated at a single species of the predicted 39-mer size, whereas the G-rich A' oligo migrated faster suggestive of an intramolecular quadruplex [21]. In addition, the A' oligo also migrated as a higher order species demonstrating intermolecular interactions which could indicate the formation of a G-quadruplex [21]. Collectively, these results demonstrate that a small G-rich telomeric sequence within the AAV ITR induces apoptosis in hESCs and lends further support that the AAV ITRs are also toxic upon AAV transduction of hESCs.

## Discussion

Our investigation using AAV vectors for the genetic modification of hESCs encountered an unexpected phenomenon: rAAV transduction directly correlated with hESC apoptosis. This phenotype was demonstrated to be independent of the viral capsid (Figures 1, 7), but rather, was attributed to the unique hESC DNA damage response elicited by the single-strand AAV origins of replication (ITR sequence), which are present on both wt and rAAV genomes. Consistently, DNA damage signaling cascades were induced upon rAAV transduction in hESCs and the apoptotic finale was dependent upon p53. The ITR dissection data from DNA microinjections supports a model in which G-rich repetitive elements of the AAV minimal origin of replication are the actual “apoptotic trigger”. The notion that this AAV “telomeric” DNA is toxic is consistent with reports of single-strand G-rich repetitive DNA at dysfunctional telomeres triggering a p53-dependent apoptotic response in other mammalian cell types [22], [23], [24]. The inability of hESCs to tolerate transduced AAV genomes is the first example of rAAV toxicity in a wild type human cell and highlights the different DNA damage responses among hESCs, particular cancers [5], and differentiated cell types.

In contrast to reports of AAV-induced toxicity in p53 deficient cancer cells [5], the work herein demonstrates the opposite scenario in hESCs; rAAV-induced toxicity is dependent upon p53. This discrepancy likely reflects the unique role of p53 in hESCs in which DNA damage induces p53-ser15 phosphorylation, p53 accumulation and p53-dependent promoter trans-activation; however some downstream effectors are not elevated at the protein level [2]. These observations were demonstrated in this work and the dramatic p53-depdendent induction of the p21 transcript upon AAV transduction did not allow detectable p21 protein. Consistently, work has demonstrated the post-transcriptional regulation of p21 protein abundance and that multiple micro RNAs, specifically expressed in pluripotent cells, target the 3′-UTR of the p21 message to down-regulate translation [4]. Another notable attribute of p53 in hESCs is the ability to induce transcription-independent apoptosis via localization to the cytoplasm and direct activation of Bax followed by cytochrome C release and caspase activation [2], [26]. These reports are consistent with the hESC response to rAAV transduction described herein, in which transcriptional induction of known apoptotic effectors was not observed, however the event was p53-dependent and abrogated by caspase inhibitors.

Within the generalized term of pluripotency there appears to be various stages which can be delineated based on their response to DNA damaging agents, the significance of which is under current investigation. This notion is supported by hESCs of different origins which display distinct properties, such as levels of the Oct4 transcript and replication indices, which directly correlate to the induction of rapid apoptosis in response to rAAV transduction or etoposide (Figures 2, S1; unpublished data). This may not be surprising as hESCs exhibit high rates of apoptosis, undergo spontaneous differentiation and multiple passages induce genomic alterations [1], [27], [28]. These observations are perhaps further illustrated by two groups who have recently reported the genetic modification of hESCs using rAAV [29], [30]. In the first instance, AAV gene correction was demonstrated in dissociated hESCs cultured in the presence of a ROCK inhibitor which has been found to pacify hESCs tendency towards apoptosis [29], [31]. The second instance is not readily explained, however, it is possible that a small fraction of hESCs demonstrate tolerance [30]. We speculate that cell differences, perhaps due to variations in the initial aliquots or perhaps maintenance of pluripotency during culture, likely account for the observed differences. Intriguingly, hESCs and murine ESCs (mESCs) also demonstrate inherently different DNA damage responses, including the presence of a debatable G1/S checkpoint [2]. In addition, mESCs undergo less spontaneous apoptosis and differentiation and are genomically stable in comparison to hESCs [32], [33]. Consistent with these reports, yet in contrast to the hESC transduction data, we, and others, have observed that mESCs tolerated AAV transduction, an (unpublished data; [25], [34]). A similarity is that by definition hESCs of different origins and mESCs are grouped under the general term of pluripotent, despite their altered response to DNA damage and predisposition for apoptosis.

Further characterization of the AAV induced DNA damage response in human pluripotent, cancer and differentiated cell types is currently underway and has strong implications for understanding the basis of oncogenesis and differentiation, as well as for the optimization of AAV as a DNA delivery vector in stem cells.

## Materials and Methods

### Cell culture conditions

Human embryonic kidney cells (293s, ATCC CRL-1573) for rAAV production, and normal human fibroblasts (NHF) were maintained at 37°C in a 5% CO_2_ atmosphere in Dulbecco's modified Eagle's medium (Sigma) supplemented with 10% fetal bovine serum and penicillin–streptomycin (100 U/ml). These experiments employ the commonly used WiCell H9 cells (hESCs unless otherwise indicated) from the National Stem Cell Bank, initially obtained from excess human embryos following *in vitro* fertilization. Cells were cultured in conditioned hESC complete medium and grown on fibronectin coated plates. The experiments herein were performed 2 days after passage at a time when hESC cell colonies are isolated and are predominantly monolayers. At this time point, Oct4 is constitutively expressed and localized within the nucleus. The hESC line H9 (WA09, XX, Passage 30–35), was cultured on feeder-free fibronectin coated plates and fed with mouse embryonic fibroblasts (mEF) conditioned human ES medium. mEFs were mitomycin-c inactivated and plated in fibroblast medium [Dulbeccos modified eagles medium (Invitrogen)], 10% fetal bovine serum (Invitrogen), 2 mM L-glutamate (Invitrogen), and 1% Penicillin/Streptomycin (Invitrogen). 24 h after attachment, the medium was changed to human ES complete medium (77% DMEM:F12 (Sigma), 20% Knockout SR (Invitrogen), 1% Non-Essential amino acids (Invitrogen), 1% Penicillin/Streptomycin (Invitrogen), 1 mM L-Glutamine (Invitrogen), 0.1 mM beta-mercaptoethanol (Sigma), 4 ng/ml basic Fibroblast Growth Factor (Invitrogen). After 24 hours, the medium was removed, filtered and used as conditioned medium for human ES cultures. Cells were cultured in 5% CO_2_ at 37°C and manually passaged every 5–6 days to maintain undifferentiated cultures. H9 hESCs were differentiated towards a neural lineage using procedure SOP-CH-207 Rev A from the national stem cell bank.

### Production of self-complementary rAAV

A previously described triple transfection method was used to generate the vectors used herein [36]. This method used the pXR series of plasmids which all contain *rep2* of AAV and individually the capsid genes of serotypes 1–9 (ex. pXR1 is p*rep2 cap1*). The phpaTRsk+ plasmid [8] was used to generate self-complementary rAAV genomes containing the *egfp* gene expressed from the CMV promoter. Following AAV production and cesium chloride gradient separation [36], fractions were analyzed for self-complementary (>90%) genomes by Southern analysis, dialyzed against PBS, and titered by quantitative PCR (Q-PCR) using the *egfp* primers: forward primer: 5′-AGC AGC ACG ACT TCT TCA AGT CC-3′ and reverse 5′-TGT AGT TGT ACT CCA GCT TGT GCC-3′.

### Production of empty particles

Production of empty and full capsids followed the rAAV production scheme described above, except that an ITR containing plasmid was not used. For these experiments AAV2 empty capsids were produced and tittered by Western dot blotting as described [36]. Membranes were probed with the primary antibody A20 [36] at 1/20 dilution in 1× PBS-0.5% tween and detection followed standard protocols [36]. It is important to note that the monoclonal A20 antibody recognizes an epitope unique to intact AAV2 capsids [37].

### Determination of intracellular transgene copy number

At 24 h post-infection cells were harvested and total DNA was purified using a Qiagen DNEasy preparation kit. This DNA then served as the template for a Q-PCR reaction using either primers for amplification of *egfp* (above) or the human lamin B gene: Forward 5′-GTT AAC AGT CAG GCG CAT GGG CC-3′ and reverse 5′-CCA TCA GGG TCA CCT CTG GTT CC-3′.

### Viral transduction

The indicated amount of rAAV particles were added in an equal volume of PBS to hESCs grown in a 24 well plate. At the indicated time points, cells were analyzed by microscopy and flow cytometry.

### Analysis of GFP+ cells

GFP+ cells were visualized by fluorescent microscopy and quantitated by flow cytometry. For flow cytometry, a minimum of 10,000 similar sized/shaped single cells that were 7-AAD negative were counted, at an event per second rate of 1,500–2,000 for each replicate at the indicated times. The GFP+ gate was designed such that untreated cells did not give a single GFP+ event beyond a million counted cells. It was also positioned away from false, or transitioning, GFP+ cells to obtain no false positives. Visualization of pooled GFP+ cells after FACs sorting confirmed the GFP+ phenotype (Flow Cytometry Core at UNC-Chapel Hill). These experiments had a N = 3 and were performed in 8 independent experiments. The standard deviation is depicted in the figure.

### Western blot

Sample preparation for Western analysis followed standard techniques [38]. Unlabeled primary antibodies against p53 (Cell Signaling), p21 (Abcam), gamma-H2AX (Millipore) and p53 phospho-ser15 (Cell Signaling) were used as recommended by the manufacturer. The secondary antibody specific for the species in which the primary antibody was generated, and labeled with Cy5, was used for visualization on a GE typhoon scanner.

### Quantitative PCR

To quantitate the transcript level of selected genes hydrolysis probes-primer set was used. The probes are provided by Roche Universal Probe Library Set, Mouse (UPL # 04 683 641 001), and Roche assay design center program was used to find ideal pair of primer and probe for each transcript (available upon request). The RT-qPCR was done on LightCycler® 480 Instrument in 10 ul of reaction volume in a 96 well plate. In each reaction 2 ul (50 ng) of cDNA, 5 ul of the probe master mix (2×), 0.5 ul of 10 uM each forward and reverse primers, 0.5 ul of 10 uM of specific probe and 2.5 ul of RNAase/DNAase free water was used. No template control was included in each run to rule out the possibility of contamination for each primer-probe set. The reaction has a hot start step at 95°C for 10 min for 1 cycle and 45 cycles of denaturation at 95°C for 10 seconds and annealing and extension at 60°C for 30 seconds. The baseline and the cp (crossing points) values were already calculated by the LC software 1.5 (Roche applied biosystem). The results were imported to the MS excel to perform relative quantitation analysis on each transcript using the delta-delta CT method. The experiments contained a N = 3 and standard deviation is depicted in the corresponding Figures.

### DNA Microinjections and oligonucleotide analysis

Human embryonic stem cells (H9) were plated onto 35 mm^2^ dishes and were microinjected on day three of culture using a Narishige micromanipulator mounted on a Leica inverted fluorescent microscope using needles pulled on a Narishige PC-10 micropipette puller. Approximately 50 hESCs were injected for each experimental condition in 2 independent experiments (note that the A and A' injections were performed only on one occasion). The microinjection buffer contained 100 mM KCl and 10 mM KPi, pH 7.4. DNA microinjections contained 100 ng/ul of a DNA oligonucleotide containing AAV ITR sequence (5′-GGCCACTCCCTCTCTGCGCGCTCGCTCGCTCACTGAGGCGCCTCAGTGAGCGAGCGAGCGCGCAGAGAGGGAGTGGCCA-3′) or a control a DNA oligonucleotide with no ITR homology (5′-GTTAGTTCACTGGGTTTATCCATATGCCAAATTGAGGGACCCAAATGTTATTTCAACTATCAATGTTATGAGCTTAGCCG-3′) as well as A (5′- GGCCACTCCCTCTCTGCGCGCTCGCTCGCTCACTGAGGC-3′) and A' (GCCTCAGTGAGCGAGCGAGCGCGCAGAGAGGGAGTGGCC). To block caspase activation, cells were pre-treated for 30 min with 50 uM zVAD-FMK (Promega), a pan-caspase inhibitor. The cells were co-injected with 5 mg/ml rhodamine dextran for visualization. Following injections, viable cells were identified as rhodamine positive and intact using phase bright microscopy. For investigation of oligonucleotide intra- and inter-molecular interactions, the A and A' 39-mers were denatured and then allowed to anneal as previously described [21]. The DNA was then electrophoresed on a non-denaturing 12% polyacrylamide gel and visualized by DNA staining.

### Immunofluorescence

To quantitate the levels of γ-H2AX (Ser 139), hESCs were treated with single-strand AAV2 particles harboring a partial *gfp* gene fragment [11] at 1,000, 10,000 and 100,000 viral genomes/cell. Cells were harvested 6 h post-infection and fixed in 95% ethanol/5% acetic acid overnight (O/N) at 4°C. Cells were washed in IFA buffer (10 mM HEPES pH 7.4, 150 mM NaCl, 4% fetal bovine serum, 0.1% NaN_3_) containing 0.5% tween 20. Cells were then re-suspended in the primary antibody staining solution (80% IFA, 0.5% tween 20, 20% DNase free RNase) with 2 ug/ml anti-H2AX phospho-Ser 139 (Millipore) FITC conjugated antibody. Following O/N incubation at 4°C, cells were washed again in IFA-0.5%-Tween 20 and resuspended in the flow analysis solution (IFA, 0.5% tween 20, 5 ug/ml RNase and 5 ug/ml propidium iodide). Single cells with 2-4N DNA content were gated on for quantitation of γ-H2AX-FITC labeling using a N = 3 in 3 independent experiments. The standard deviation is presented in the Figure 2B.

For annexin V staining, hESCs were pre-treated with a caspase inhibitor prior to infection with 10,000 particles of scAAV3B-CMV-*egfp*. 8 h post-infection cells were harvested and stained with an annexin V – Alexafluor 647 conjugate according to the manufacturer's instructions (Invitrogen). The degree of labeling was determined by flow cytometry using a N = 3 on 2 different occasions. The standard deviation is depicted in the figure.

### Flow Cytometry Cell Cycle Analysis

Dividing hESCs were treated with 200 ug/mL nocodazole and 10 uM EdU was added to each sample 2 h before harvest. Samples were harvested 0, 2, 4, or 6 h after nocodazole addition with TrypLE (Invitrogen, Carlsbad, CA) and cells were pelleted by centrifugation. All samples were fixed in 95% ethanol/5% acetic acid, and stained with a Cy5-labeled MPM-2 primary antibody (Millipore, Billerica, MA) to identify mitotic cells [35]. To determine S phase fractions, EdU was detected using a Click-It EdU-488 flow cytometry kit (Invitrogen, Carlsbad, CA) according to manufacturer's instructions. DAPI was used to measure DNA content. Samples were measured on a Dako CyAN ADP instrument at the Flow Cytometry Core Facility at UNC-CH. Flow cytometry samples were analyzed using Summit 4.3 software to quantify the percentage of hESCs with 4N DNA content that were also labeled with MPM-2, a specific marker of mitosis. The percentage of mitotic cells for each sample was plotted against time and the resulting slope of the line was used to measure the rate of entry into mitosis (the percentage of hESCs entering mitosis per hour). hESCs that incorporated EdU and contained 2N-4N DNA content were measured as the S phase fraction. Results are an average of three independent experiments in each hESC cell line and the standard deviation is depicted on the graph.

## Supporting Information

Figure S1 **Chromosome condensation in rAAV treated hESCs.** Chromosome condensation was evaluated by DAPI staining to determine whether the hESCs were undergoing apoptosis in response to AAV infection. Eight hours after scAAV3B infection, hESCs treated with 100,000 scAAV3B-*egfp* particles demonstrated a dramatic increase in punctate nuclear staining compared to cells that were not treated.
(EPS)

Figure S2 **Recombinant AAV Transduction of hESC WiCell H1s.** **A**) WiCell H1s were transduced with 100,000 particles of scAAV3B-CMV-*egfp*. The cells were harvested for GFP analysis by flow cytometry 24 h after virus addition. B) Cell viability was measured by dye exclusion at the indicated timepoints following transduction of H1 cells by 100,000 scAAV3B-CMV-egfp particles. C) Total RNA was harvested from H9 or H1 hESCs and used as a template for reverse transcription. cDNA was investigated by Q-PCR to determine the amount of Oct4 transcript in each line normalized to the housekeeping transcript GAPDH. D) Rates of mitotic entry for H1 and H9 hESCs. Dual color flow cytometry using a mitosis-specific antibody to phospho-MPM2 epitope (Cy5) and DAPI to analyze DNA content was employed to measure mitotic entry rates. Samples were subjected to nococdazole treatment for 2, 4, or 6 hours. Mitotic entry rates were calculated as linear regression slopes generated from scatter plots of the percentage of MPM2+ cells over time. (* indicates p-value<0.005).
(EPS)

Figure S3 **Recombinant AAV Transduction of H9 cells Differentiated Towards a Neural Lineage.** A) hESCs were grown to embryoid bodies and differentiated towards a neuronal lineage for 15 days. Then, 100,000 scAAV3b-CMV-*egfp* particles were used for transduction and viability was determined 24 h later by dye exclusion. B) Immuno-fluorescence of cells following scAAV3B-CMV-*egfp* transduction using an anti-vimentin antibody and the native eGFP fluorescence.
(TIF)

Figure S4 **Characterization and Transduction of p53 deficient hESCs.** (A) Equal amounts of protein from wild type H9 hESCs and those knocked down for p53 transcript [described in (B)] were analyzed for total p53 abundance by Western blotting. Alpha-tubulin served as additional loading controls. (B) Wild type or p53 knockdown hESCs were analyzed for the Oct4 transcript by reverse transcriptase followed by quantitative PCR using cDNA template. The values were normalized to levels of the housekeeping transcript GAPDH. Results are presented as transcript induction which is the value determined for AAV infected cells divided by the value determined for the no treatment group (p-value>0.5). (C) H9 hESCs deficient for p53 were transduced with transduced by rAAV3B-gfp (100,000 viral genomes/cell) and images of colony integrity and GFP fluorescence are provided at the time points following transfection.
(EPS)

Figure S5 **Densitometry Analysis of Western Blots.** The indicated Western blotting experiments were analyzed for a relative abundance using a storm scanner and Image Quant 5.2. Internal loading controls were used for normalization. (ND indicates no signal detected and NT represents no treatment).
(EPS)

Figure S6 **Transcript Abundance of Apoptotic Effectors.** H9 hESCs were treated with rAAV3B-CMV-egfp (1e5 viral genomes/cell) or equal volume of PBS. RNA from treated cells was harvested, converted to cDNA and copy number of the indicated transcripts was determined by Q-PCR. Lamin B2 transcript abundance was used for normalization to cell number and the data is presented as the normalized transcript abundance of the rAAV treated cells divided by the vehicle control. In all cases the transcript abundance change in cells treated with AAV was not significantly different than the non-treated (NT) controls (p-value>0.2).
(EPS)

Figure S7 **Quantitation of the DNA Microinjection Experiment.** For each of the indicated injection regimens approximately 100 hESCs were injected with the indicated DNA oligonucleotide (oligo) as described in the results and methods. Two hours post-injection rhodamine positive cells were tallied and are presented as a percentage of the total injected. (* indicates p-value<0.005).
(EPS)

## References

[1] Dravid G, Ye Z, Hammond H, Chen G, Pyle A (2005). Defining the role of Wnt/beta-catenin signaling in the survival, proliferation, and self-renewal of human embryonic stem cells.. Stem Cells.

[2] Qin H, Yu T, Qing T, Liu Y, Zhao Y (2007). Regulation of apoptosis and differentiation by p53 in human embryonic stem cells.. J Biol Chem.

[3] Grandela C, Pera MF, Wolvetang EJ (2007). p53 is required for etoposide-induced apoptosis of human embryonic stem cells.. Stem Cell Res.

[4] Wang Y, Blelloch R (2009). Cell cycle regulation by MicroRNAs in embryonic stem cells.. Cancer Res.

[5] Raj K, Ogston P, Beard P (2001). Virus-mediated killing of cells that lack p53 activity.. Nature.

[6] Morita E, Nakashima A, Asao H, Sato H, Sugamura K (2003). Human Parvovirus B19 Nonstructural Protein (NS1) Induces CellCycle Arrest at G1 Phase.. J Virol.

[7] Lilley CE, Schwartz RA, Weitzman MD (2007). Using or abusing: viruses and the cellular DNA damage response.. Trends Microbio.

[8] McCarty DM, Monahan PE, Samulski RJ (2001). Self-complementary recombinant adeno-associated virus (scAAV) vectors promote efficient transduction independently of DNA synthesis.. Gene Ther.

[9] Hewitt FC, Samulski RJ (2010). Creating a novel origin of replication through modulating DNA-protein interfaces.. PLoS One.

[10] Samulski RJ, Chang LS, Shenk T (1987). A recombinant plasmid from which an infectious adeno-associated virus genome can be excised in vitro and its use to study viral replication.. J Virol.

[11] Choi VW, McCarty DM, Samulski RJ (2006). Host cell DNA repair pathways in adeno-associated viral genome processing.. J Virol.

[12] Inagaki K, Ma C, Storm TA, Kay MA, Nakai H (2007). The role of DNA-PKcs and artemis in opening viral DNA termini in various tissues in mice.. J Virol.

[13] Cervelli T, Palacios JA, Zentilin L, Mano M, Schwartz RA (2008). Processing of recombinant AAV genomes occurs in specific nuclear structures that overlap with foci of DNA-damage-response proteins.. J Cell Sci.

[14] Schwartz RA, Palacios JA, Cassell GD, Adam S, Giacca M (2007). The Mre11/Rad50/Nbs1 complex limits adeno-associated virus transduction and replication.. J Virol.

[15] Fragkos M, Jurvansuu J, Beard P (2009). H2AX is required for cell cycle arrest via the p53/p21 pathway.. Mol Cell Biol.

[16] Gartlel AL, Radhakrishnan (2005). Lost in transcription: p21 repression, mechanisms and consequences.. Cancer Res.

[17] Barta T, Vinarsky V, Holubcova Z, Dolezalova D, Verner J (2010). Human embryonic stem cells are capable of executing G1/S checkpoint activation.. Stem Cells.

[18] Neganova I, Vilella F, Atkinson SP, Lloret M, Passos JF (2011). An important role for CDK2 in G1 to S checkpoint Activation and DNA damage response in human embryonic stem cells.. Stem Cells.

[19] Johnson JS, Samulski RJ (2009). Enhancement of adeno-associated virus infection by mobilizing capsids into and out of the nucleolus.. J Virol.

[20] Keniry MA (2000–2001). Quadruplex structures in nucleic acids.. Biopolymers.

[21] Oganesian L, Moon IK, Bryan TM, Jarstfer MB (2006). Extension of G-qaudruplex DNA by Ciliate Telomerase.. EMBO.

[22] Karlseder J, Broccoli D, Dai Y, Hardy S, de Lange T (1999). p53- and ATM-dependent apoptosis induced by telomeres lacking TRF2.. Science.

[23] Campisi J, Kim SH, Lim CS, Rubio M (2001). Cellular senescence, cancer and aging: the telomere connection.. Exp Gerontol.

[24] Cosme-Blanco W, Shen MF, Lazar AJ, Pathak S, Lozano G (2007). Telomere dysfunction suppresses spontaneous tumorigenesis *in vivo* by initiating p53-dependent cellular senescence.. EMBO.

[25] Smith-Arica JR, Thomson AJ, Ansell R, Chiorini J, Davidson B (2003). Infection efficiency of human and mouse embryonic stem cells using adenoviral and adeno-associated viral vectors.. Cloning Stem Cells.

[26] Chipuk JE, Kuwana T, Bouchier-Hayes L, Droin NM, Newmeyer DD (2004). Direct activation of Bax by p53 mediates mitochondrial membrane permeabilization and apoptosis.. Science.

[27] Ezashi T, Das P, Roberts RM (2005). Low Oxygen tensions and the prevention of differentiation of hES cells.. Proc Natl Acad Sci U S A.

[28] Maitra A, Arking DE, Shivapurkar N, Ikeda M, Stastny V (2005). Genomic alterations in cultured human embryonic stem cells.. Nat Genet.

[29] Mitsui K, Suzuki K, Aizawa E, Kawase E, Suemori H (2009). Gene targeting in human pluripotent stem cells with adeno-associated virus vectors.. Biochem Biophys Res Commun.

[30] Khan IF, Hirata RK, Wang PR, Li Y, Kho J (2010). Engineering of human pluripotent stem cells by AAV-mediated gene targeting.. Mol Ther.

[31] Watanabe K, Ueno M, Kamiya D, Nishiyama A, Matsumura M (2007). A ROCK inhibitor permits survival of dissociated human embryonic stem cells.. Nat Biotechnol.

[32] Corbet SW, Clarke AR, Gledhill S, Wyllie (1999). p53-dependent and independent links between DNA-damage, apoptosis and mutation frequency in ES Cells.. Oncogene.

[33] Hong Y, Stambrook PJ (2004). Restoration of an absent G1 arrest and protection from apoptosis in embryonic stem cells.. Proc Natl Acad Sci USA.

[34] Henckaerts E, Dutheil N, Zeltner N, Kattman S, Kohlbrenner E (2009). Site-Specific integration of adeno-associated virus involves partial duplication of the target locus.. Proc Natl Acad Sci USA.

[35] Bower JJ, Karaca GF, Zhou Y, Simpson DA, Cordeiro-Stone M (2010). Topoisomerase IIalpha maintains genomic stability through decatenation G(2) checkpoint signaling.. Oncogene.

[36] Grieger JC, Choi VW, Samulski RJ (2006). Production and characterization of adeno-associated viral vectors.. Nat Protoc.

[37] Wobus CE, Hugle-Dorr B, Girod A, Petersen G, Hallek M (2000). Monoclonal antibodies against the adeno-associated virus type 2 (AAV-2) capsid: epitope mapping and identification of capsid domains involved in AAV-2-cell interaction and neutralization of AAV-2 infection.. J Virol.

[38] Hirsch ML, Storici F, Li C, Choi VW, Samulski RJ (2009). AAV recombineering with single strand oligonucleotides.. PLoS One.

