# Supplementary figures and images for: Viral Single-Strand DNA Induces p53-Dependent Apoptosis in Human Embryonic Stem Cells
Source: PLoS One. 2011 Nov 17;6(11):e27520. doi: 10.1371/journal.pone.0027520 (PMC3219675; doi:10.1371/journal.pone.0027520)

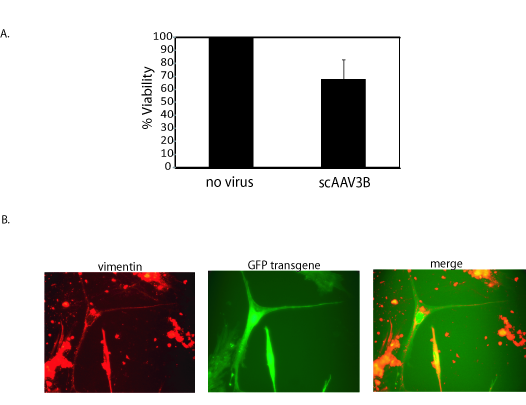

Supplement: Figure S3 — Recombinant AAV Transduction of H9 cells Differentiated Towards a Neural Lineage. A) hESCs were grown to embryoid bodies and differentiated towards a neuronal lineage for 15 days. Then, 100,000 scAAV3b-CMV-egfp particles were used for transduction and viability was determined 24 h later by dye exclusion. B) Immuno-fluorescence of cells following scAAV3B-CMV-egfp transduction using an anti-vimentin antibody and the native eGFP fluorescence. (TIF) [file pone.0027520.s003.tif]
